# Provider Practices and Perceived Barriers and Facilitators in Improving Quality Practices in Radiation Oncology Peer Review

**DOI:** 10.1016/j.adro.2024.101708

**Published:** 2025-01-08

**Authors:** Leslie Chang, Sara Alcorn, Khinh Ranh Voong, Todd R. McNutt, Ori Shokek, Suzanne Evans, Jean L. Wright

**Affiliations:** a Department of Radiation Oncology, University of Minnesota, Minneapolis, Minnesota; b Department of Radiation Oncology and Molecular Radiation Sciences, Johns Hopkins University School of Medicine, Baltimore, Maryland; c Wellspan Radiation Oncology, York, Pennsylvania; d Department of Radiation Oncology, Yale School of Medicine, New Haven, Connecticut; e Department of Radiation Oncology, University of North Carolina, Chapel Hill, North Carolina

## Abstract

**Purpose:**

Radiation oncology peer review evaluates case-specific qualitative treatment planning decisions. We sought to understand interdisciplinary perspectives on peer review to identify factors affecting stakeholder engagement and implementation of recommendations.

**Materials and Methods:**

Semistructured interviews and Likert surveys (scaled, 0-10) with radiation oncology peer review participants were audio-recorded and transcribed. Two independent coders utilized a grounded theory approach to extract dominant themes.

**Results:**

Participants included 6 academic and 3 community radiation oncologists, 2 residents, 2 medical physicists, 2 radiation therapists, 4 dosimetrists, and 1 industry representative. Thematic priorities of peer review included adherence to institutional guidelines, clinical background to inform decision-making, detection of rare errors, and education. Key facilitators included pretreatment peer review, clear planning guidelines, and feedback on peer recommendations. Barriers to recommendation adoption included resource limitations and a lack of prospective data guiding qualitative recommendations. Participants perceived benefits of peer review were assessed with Likert surveys with higher values placed on reducing practice variation (8.0) and education (7.6) and a lower value placed on the detection of medical errors (7.4) and reduction of treatment delivery incidents (6.9). When comparing Likert scores by participant role, nonphysicians rated the overall importance of peer review (mean, 9.8 vs 6.5, *P* = .03) and education (mean, 9.0 vs 6.7, *P* = .02) significantly higher than physicians.

**Conclusion:**

Participants in radiation oncology acknowledged the importance of peer review, but there was significant variation in the perceived benefits. A higher value was placed on the alignment of clinical practice and nonphysician participant education. Future processes to improve communication and prospective plan review were identified as beneficial to peer review-mediated plan changes.

## Background

Peer review of patient treatment plans has been established as an important aspect of quality assurance (QA) in radiation oncology and is required for accreditation in professional organizations such as the American Society for Radiation Oncology, the American College of Radiation Oncology, and the American College of Radiology.^1^, ^2^, ^3^ The seminal American Society for Radiation Oncology white paper reaffirmed the role of peer review, also commonly titled “chart rounds,” as a mechanism for discussion of qualitative decisions to optimize radiation treatment plans that “often do not have a clear right or wrong answers,”^4^ and therefore metrics to define improvements based on peer review are difficult to develop. Other QA mechanisms are in place to detect quantitative errors in treatment plans. Thus, there is debate as to the greatest impacts of peer review, with a range of potential outcomes, including adherence to plans with existing departmental or national planning goals, reduction in variation in practice, education, and detection of medical errors.^5^ Peer review has the potential to improve treatment outcomes by improving guideline-concordant care and identifying errors. Significant deviation from radiotherapy protocols (eg, undetected or uncorrected deviations from best practice) has been shown to decrease overall survival.^6^^,^^7^ However, reported rates of plan changes after peer review programs range widely, with changes in up to a quarter of cases.^8^^,^^9^

Peer review is one method of audit and feedback used to improve professional practice; however, the characteristics that lead to successful feedback interventions and quality improvement of radiation treatment plans are unknown. A systematic review of the impact of feedback has demonstrated that the quality of data, motivation, and interests of health care providers and organizational support for QA are important effect modifiers.^10^ Prior qualitative studies have found that physicians desire and value feedback and have substantive suggestions on methods to improve feedback presentation and formatting to make them more useful, meaningful, and actionable to improve patient care.^11^ It is imperative to understand the peer review user perspective to meaningfully increase buy-in from health care providers, improve the effectiveness of peer recommendations, and understand factors prompting plan modification. Additionally, evaluation of the barriers to effective peer review can allow for targeted approaches leveraging information technology and personnel to decrease reviewer time and associated costs. Peer review is often an interdisciplinary conference attended by physicians, trainees, as well as physics, dosimetry, and therapy colleagues, and little is known about their perceptions and participation in this quality improvement program.^4^ We hypothesized that diverse stakeholder groups may perceive the benefits of peer review differently and have novel barriers to participation. Our objective is to identify the aspects of peer review that meet the needs and goals of participants in order to improve plan quality and safety through peer recommendations and accuracy in plan changes.

## Materials and Methods

### Overview

This mixed-methods study explored the experiences and perspectives of participants in radiation oncology peer review, focusing on understanding interdisciplinary views to identify factors influencing stakeholder engagement and implementation of peer review recommendations.

### Study population

A convenience sample of participants from unique practices was identified through membership in radiation oncology organizations, prior working relationships with the oncologists, or referrals from other participants. A sample size of ≤25 participants was planned, and enrollment was continued until thematic saturation was reached. Qualitative interviews were conducted with 6 key stakeholder groups, including those in academic and community practice: (1) radiation oncologists, (2) resident radiation oncologists, (3) dosimetrists, (4) industry peer review software designers, (5) radiation therapists, and (6) medical physicists. This work was conducted with a waiver of informed consent under Johns Hopkins institutional review board approval.

### Data collection

All participant interviews were audio-recorded and transcribed using a virtual Zoom platform. Interviews utilized a semistructured interview guide focused on the perceived goals of peer review (Appendix E1). During the interview, participants were asked to quantify the greatest value from peer review using Likert scoring (0-10, eg, “not important,” 0, and “extremely important,” 10) to determine the greatest value from peer review from 6 choices: improvement of treatment planning processes, adherence of plans with institutional guidelines, reduction of variation in practice, education, reduction of treatment delivery incidents (ie, inaccurate patient setup on the machine), and detection of medical errors. Three cases were adapted from the Radiation Oncology Incident Learning System,^12^ which involved errors discovered in peer review (target volume errors with a missing clip, normal structure tolerance dose exceeded, and wrong site treatment in a 2 isocenter case). These cases included a mock treatment plan and peer review suggestions and were used as an inductive strategy for our grounded theory qualitative approach. Our participants discussed potential barriers to treatment plan modification, possible facilitators, and personal experiences with the peer review process.

### Data analysis

Two independent coders (LC and SA) reviewed transcripts for this analysis and generated an analytic codebook based on a priori and emergent codes using MAXQDA version 22.6. Codes were reviewed in an iterative fashion every 4 transcripts using a constant comparative method to determine when coding saturation was reached and to ensure coding consistency. Discrepancies were reviewed and discussed until a consensus was reached. Once all coders felt that thematic saturation had been reached, the final codebook was reviewed and agreed upon by all coders. Thematic or coding saturation is defined when no additional themes or insights emerge from the data collection and all conceptual categories have been explored, identified, and described.^13^ For quantitative analysis ANOVA and Student *t* test were used to analyze Likert scoring.

## Results

A total of 20 participants participated in qualitative interviews with balanced representation between males versus females (50% vs 50%) and between physicians and nonphysicians (55% vs 45%) (Table 1**)**. Physician participants were distributed across 6 practices, with the majority practicing in academic centers (80%). Nonphysician participants were primarily from 1 to 2 academic centers (89%). Participation from dosimetrists and physicists during plan review was highlighted as important to attendees but not mandatory to the peer review process. The discussion always includes a presentation of the patient's clinical characteristics, treatment approach, prescription, and treatment plan.^4^^,^^14^ The majority of peer review conferences took place virtually, although there could be some individuals who meet in a conference room with virtual meeting capabilities. Additional content of peer review meetings differed among participants, and some but not all meetings are disease site-specific, evaluate if patients are candidates for clinical trials, and include a review of patient setup imaging.

**Table 1:** Participant characteristics

Characteristic	Category	N = 20 (%)
Gender	Female	10 (50%)
	Male	10 (50%)
Professional background	Attending physician	9 (45%)
	Resident physician	2 (10%)
	Medical physicist	3 (15%)
	Dosimetrist	4 (20%)
	Therapist	2 (10%)
Professional location	Academic	16 (80%)
	Community	3 (15%)
	Industry software developer	1 (5%)

**Figure 1:**
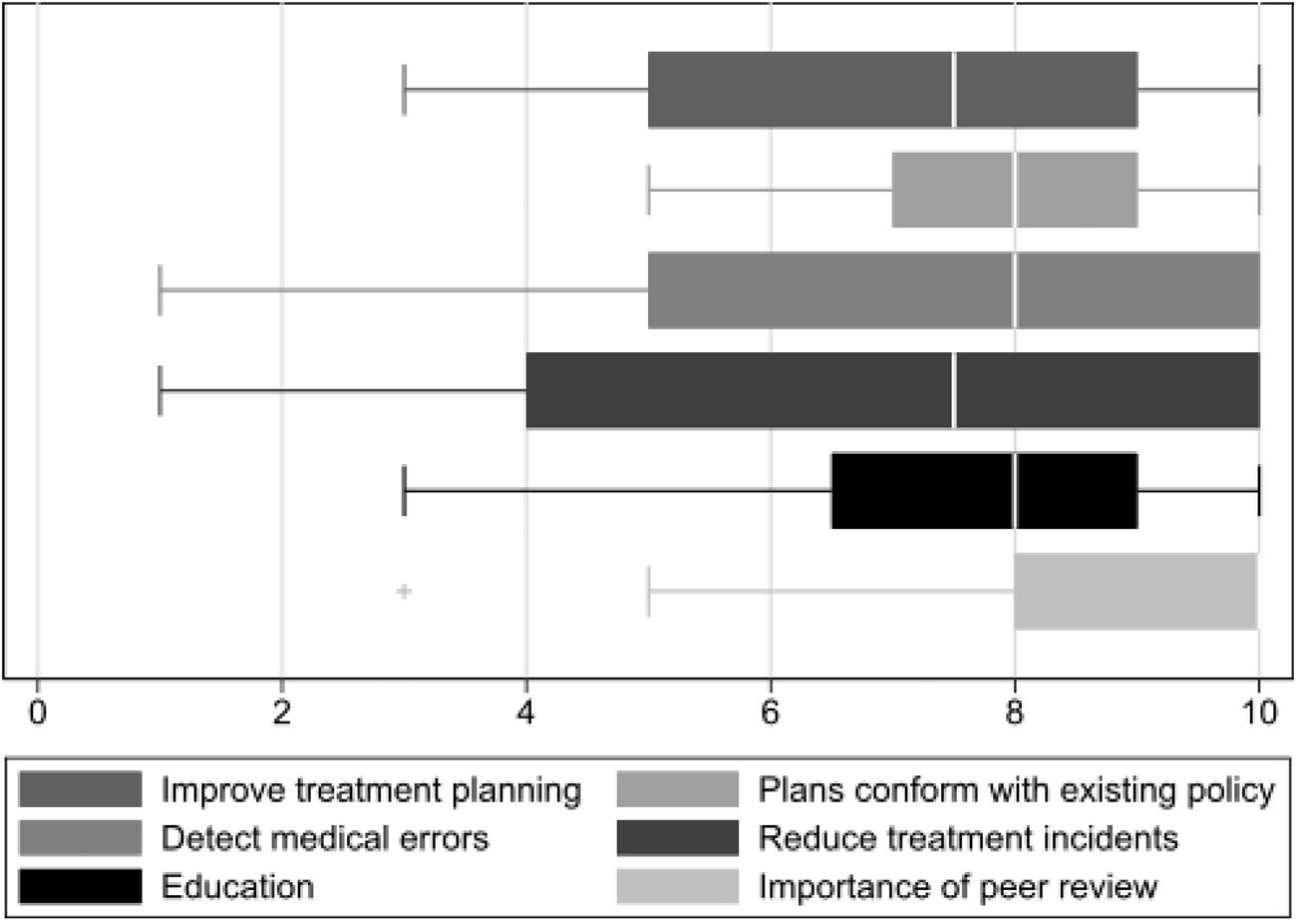
Likert survey regarding the goals of peer review. The range (horizontal bar), interquartile range (box), and median score (white vertical line) are illustrated.

The respondents were surveyed regarding their goals in attending the peer review conference using a Likert scale (Fig. 1). The majority of participants rated peer review as an important aspect of QA, scoring 8.2 out of 10. In order of importance, participants rated adherence to plans with institutional guidelines (mean, 7.8, SD, 1.3) and participant education (mean, 7.6, SD 2.4), followed by the detection of medical errors (mean, 7.4, SD, 2.7), improvement of treatment planning (mean, 7.2, SD, 2.3), and reduction of treatment delivery incidents, (mean, 6.9, SD, 3.1). When comparing Likert scores by role, nonphysicians rated the overall importance of peer review (mean, 9.8 vs 6.5, *P* = .03) and education (mean, 9.0 vs 6.7, *P* = .02) significantly higher than physicians. Although not statistically significant because of sample size, there was no appreciable difference in Likert scoring between academic and nonacademic participants.

Four key themes emerged for the importance of peer review: (1) adherence to institutional guidelines, (2) education of providers and trainees, (3) clinical background to inform decision-making, and (4) detection of rare errors (Table 2). The first was the concept that peer review improves coherence in practice. All participants agreed that peer review is important for patients to receive the same standard of care and align treatment recommendations despite differences in physician practices. Some practices had extremely standardized practices where dose and fractionation for disease sites were clearly stated depending on the volume being treated, whereas others had more variability. In summary, peer review is seen as an important audit of treatment plan recommendations to ensure patients receive the same standard of care in concordance with clinical guidelines.

**Table 2:** Emergent themes of peer review

Theme	Participant profession	Descriptive and illustrative quotes
Adherence to institutional guidelines	Attending physician	“I would not want a patient, (to) come to see me and have me say it would be unacceptable to hypofractionation in your case, and then go see my colleague who sits across the hall from me and have her say it is completely reasonable.”
	Medical physics	“Using peer review to help maintain policy is appropriate and should be checked.”
	Dosimetrist	“Peer review is important in reducing (physician) variation. I have suggested to leadership that we have a standard (that) the plans have to meet for each disease site.”
Education of providers and trainees	Attending physician	“Our group really felt comfortable with hypofractionation in a very similar case and that helps me feel more confident that really is a good approach for this case that I am working on now.”
	Attending Physician	“Because we are pretty emphatic that we should not rush through cases. And if someone has an interesting tidbit or a question about new studies that suggest doing it one way or another. I think that is very important to discuss in chart review (and we) permit time for that.”
	Attending physician	“(Site-specific peer review) is not that much educational value because questions are not asked by people who are unfamiliar with current treatment approaches, and so there are fewer questions.”
	Attending physician	“Chart rounds are so busy with so many cases to present that if there is something that gives it tends to be (…) Socratic style questioning of the residents.”
	Medical physicist	“(Physics residents) are involved pretty deeply in each case but that is the 1 case. Peer review gives (them) exposure to the rest of the experiences of the clinic and often cases that require difficult clinical decision making.”
	Medical physicist	“I learned a lot and understand the viewpoint of physicians on each patient case and those discussions are very helpful.”
	Dosimetrist	“I have no idea why we are giving these brain mets 3 fractions in 21 Gy while this (other) one is getting 18 Gy. I would love to hear the interactions back with the physicians.”
Clinical background to inform decision-making	Resident physician	“I think having someone there who can explain the clinical scenario would help at times when it is unclear, what the motivation and intent was”
	Attending physician	“Well, maybe there is […] a critical structure in close enough proximity to the target volume or the dose goal limit for that structure would be exceeded. We may come to the conclusion that it is worth sacrificing coverage of the planning volume in order to have a safer dose to the critical structure.”
	Attending physician	“I talked to the patient about the risks and benefits for this high dose to the airway at risk and they were on board with it, (…) We all understand it was a potentially toxic treatment, but we felt like it's the best way to treat this.”
Detection of rare errors	Radiation therapist	“So I think it is a really low probability that a clinically significant error would happen but it is definitely possible and I think (more common) for more complicated cases or stereotactic cases. I think about those (in peer review) all the time.”
	Medical physicist	“The medical error that we are trying to find is the needle in the haystack, but it is worth our while.”

A second key theme was education for physician and nonphysician participants. Physicians varied in their belief in the educational value of peer review conferences, particularly those who practiced in academic institutions. Physicians in academic institutions described some aspects of peer review that limit the educational value, which included the time stress of presenting and reviewing all the patient cases in the allotted time (Table 2). This can decrease educational value, especially for trainees where there is a lack of defined expectations for education. In addition, some academic programs have disease site-specific peer reviews, which can be less educational because physicians are very familiar with current treatment approaches, decreasing the opportunity for discussion. However, other academic and nonacademic physicians described how they adopted new practice patterns from peer review discussions, and allowing time to discuss cases encouraged learning from colleagues. This allowed for increased confidence in new techniques and prescriptions such as proton therapy and hypofractionation. In contrast, nonphysicians derived significant educational benefits from understanding the physician's clinical judgment during peer review and learning planning techniques from other participants. Nonphysicians described benefits to trainees, such as medical physics residents, because peer review can broaden the types of cases observed and increase exposure to clinical decision-making.

The third theme was clarifying the clinical scenario to put the treatment planning directives in context. Patient-specific clinical history affects treatment decision-making, and all participants agreed this is an important topic discussed in peer review. In addition, the physician's treatment intent and prescription to the clinical target volume are derived from these characteristics. Both attending and resident physicians emphasized the importance of physician presence at peer review to describe their intent in certain decision-making, such as the tradeoff between target coverage versus sparing organs-at-risk. Peer review can also assist in reviewing plans that exceed constraints in the context of curative or life-prolonging therapy without other reasonable treatment alternatives after shared decision-making with the patient.

The fourth theme was the importance of peer review to detect rare errors. Overall QA processes to catch medical errors are very important; however, many physician and nonphysician participants clarified that catching these errors during peer review is uncommon as there are multiple other QA processes in place to detect errors before a treatment begins. Nonphysician participants, including medical physicists, dosimetrists, and radiation therapists, highlighted the multiple other safety checks performed, including confirming laterality, isocenter coordinates, and consistency in the prescription. However, most individuals agreed that all processes to catch errors are critically important, including peer review, because 1 medical physicist described: “The medical error that we are trying to find is the needle in the haystack, but it is worth our while.”

### Facilitators and barriers to peer review-mediated plan changes

**Figure 2:**
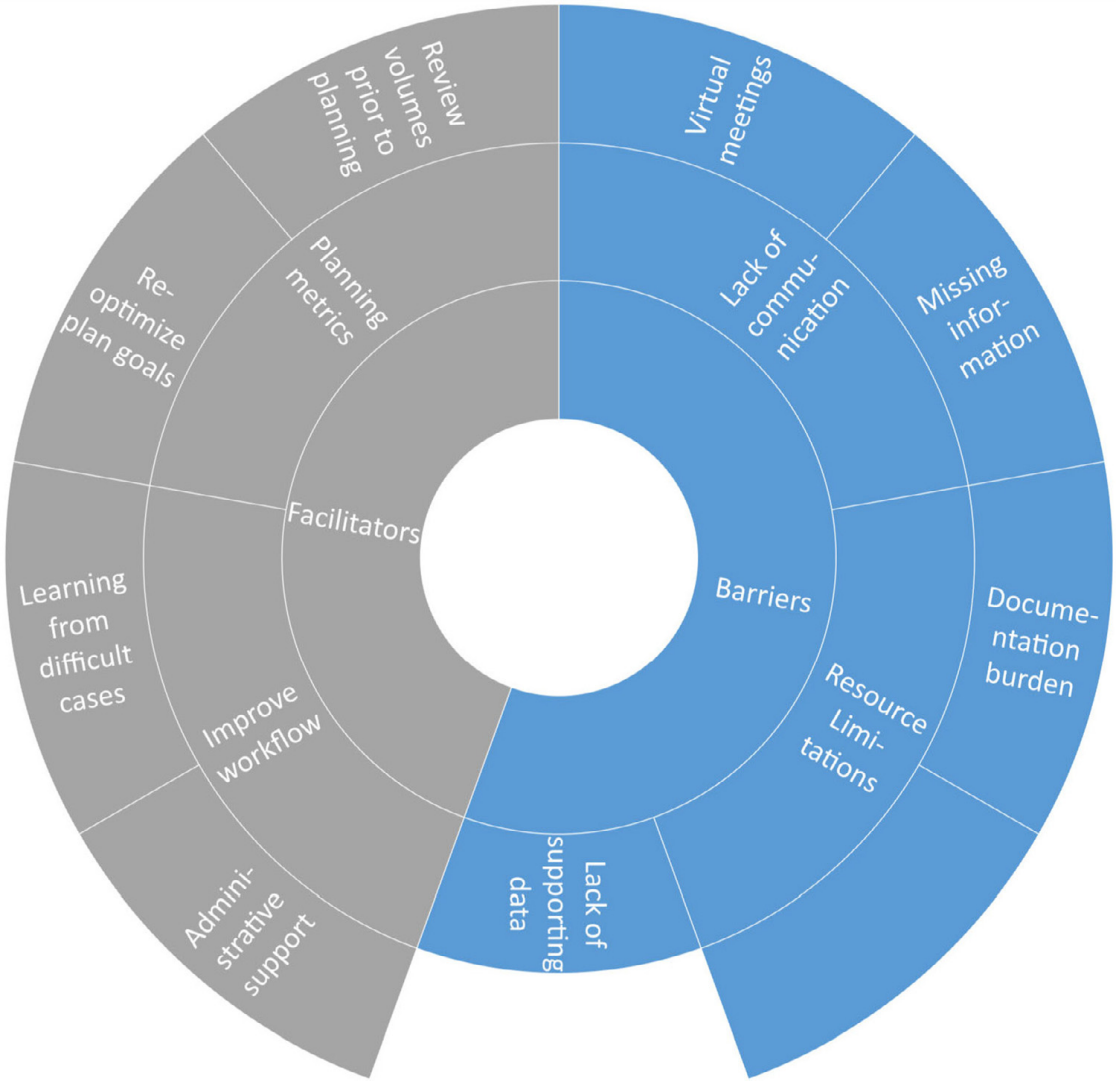
Facilitators and barriers for peer review. The sunburst diagram represents the qualitative results. Within the barriers and facilitator themes (inner ring), sub themes (middle ring) are assigned and specified (outer ring).

Participants were able to identify several aspects of peer review that facilitate treatment plan changes, including specific identifiable planning metrics as well as workflow improvement **(**Fig. 2). For example, in a reirradiation setting, when a plan exceeds a maximum dose constraint to a particular organ-at-risk, participants will recommend reoptimization of the plan. In addition, the timing of peer review is important. Review of the clinical target and organs-at-risk volumes prior to treatment planning and/or treatment starting in peer review or contour review conferences was identified as beneficial to identify plans for greater discussion and give the planning team time to make changes. Multiple participants also identified technological and personnel support as important to peer review practices. Some examples included support for recording and feedback for peer review recommendations **(**Table E1**)**. The software industry participant described methods to improve efficiencies within peer review, including programs to allow the recording of peer review performed outside of set conference times and without the difficulties of switching between multiple software platforms. Multiple nonphysician participants have also described the need to adopt a peer review workflow to follow-up on peer review recommendations as an avenue of innovation to facilitate recommended plan changes.

Barriers to peer review recommendations focused on difficulty in communication due to lack of participation, lack of prospective data for opinion-based recommendations, and resource limitations to implement changes (Fig. 2). Many participants described concern for decreased participation in peer review because of virtual conferences (Table E1). Sometimes there is no clear data behind peer review recommendations, and it is unclear whether recommendations are opinion-based rather than adherence to a standard of care. These nuances in clinically acceptable planning can be difficult to assess. For example, multiple dosimetrists discussed specific dosimetric objectives that may be limited to the capabilities of the treatment planning system, and small changes may not result in clinical differences. Attending physicians and dosimetrists also commented on the burden of time, both for peer review as well as replanning based on recommendations. This can result in physician plan change hesitancy because of fear of inconveniencing others. As 1 attending physician describes: “This plan has already been generated. You are resistant to any change at this point. You feel bad for your planner for having to redo all the work that they already did because you made a silly mistake.” Multiple dosimetrists also describe how time constraints in replanning can lead to more errors in order to not delay patients starting or continuing their course of treatment. Time stress is a key barrier to plan modification. Other resource limitations include the time constraints for peer review because physician and nonphysician participants have described a lack of time for clinical care because of attendance at multiple meetings throughout the day and additional clinical documentation requirements. Peer review meetings should continue to improve communication between participants to clarify poor quality versus stylistic plan recommendations and implement workflows that can streamline plan changes, such as prospective peer review or recorded peer review outside of designated times.

## Discussion

Our study describes interdisciplinary perspectives on peer review in radiation oncology and identifies 4 main themes that influence stakeholder engagement and implementation of peer review recommendations: adherence to institutional guidelines, education of providers and trainees, giving clinical background to inform decision-making, and the detection of rare errors. Additionally, clear institutional planning metrics, feedback for peer recommendations, and prospective treatment plan reviews facilitated peer review-mediated plan changes. Our findings highlighted the nuanced, qualitative aspects of the peer review process and the inherent challenges in defining the benefits and optimal structure for peer review in this context.

An aspect of peer review that remains a challenge to physician and nonphysician participants includes the time stress of implementing recommendations and the lack of a structured system for feedback on plan changes. One potential solution includes pretreatment or prospective peer review, which has been found to increase the percentage of recommendations,^15^ likely because of increased detail in review and time to make changes. Prospective plan review can include peer review of clinical workup and reviewing target volumes on treatment planning software prior to treatment plan completion. Implementation of pretreatment segmentation and plan review has been successful in several institutions, with improvement in quality through higher implementation rates of peer recommendations and a decrease in the percentage of major errors identified over time.^16^, ^17^, ^18^, ^19^ The decision to perform contour and segmentation peer review prior to dosimetric planning differs between institutions and may depend on several factors, including definitive versus palliative treatment, disease site, technical complexity (reirradiation, involvement of critical organs-at-risk, type of technology used [3-dimensional vs intensity modulated radiation therapy, brachytherapy, proton, or stereotactic radiation therapy]). In future implementation studies, methods should correlate the timing of peer review from treatment start with the percentage of peer review-mediated plan changes to better understand provider workflow and which planning factors derive the most benefit from prospective review. These results will complement longitudinal studies to study the clinical outcomes and concordance of peer review recommendations with clinical guidelines.

There are new concerns about the lack of engagement with a virtual peer review format, with decreases in both participant discussion and suggested plan revisions.^20^^,^^21^ Although virtual meetings allow for greater engagement with participants working remotely or in different clinical locations, the tradeoff in terms of engagement was noted by multiple stakeholders. Future methods to improve these difficulties are being studied with the implementation of new technological tools to automate aspects of case review, such as preloading the appropriate cases and simple documentation of peer scoring or commentary.^22^ decreasing time spent in each meeting. In addition, machine learning algorithms can assist in flagging cases with abnormal dose constraints or contours for further review.^23^ Furthermore, protecting meeting time, such as not scheduling simulations during meetings, improves attendance in chart rounds^24^ and allows peers to focus on case review. Importantly, investment into personnel and technology to assist in peer review and follow-up on plan changes is critical for continued QA engagement.

This study has potential limitations. Our sampling did include an interdisciplinary peer review group; however, the majority of participants practiced in academic centers. This may inadequately represent participants from community practices. Additionally, we had a smaller sampling of some nonphysician participants, including medical physicists and radiation therapists, which may not encompass the breadth of their experience in the peer review process. Some nonphysician participants do not routinely participate in peer review and therefore were unable to participate in the study. However, given the educational benefits nonphysicians derive from the peer review process, their involvement should be encouraged whenever possible. The inclusion of a larger sample of radiation oncology peer review participants with more varied backgrounds could provide additional insights.

Radiation oncology peer review is a unique aspect of the field, allowing for prospective discussion of radiation treatment plans, either before treatment has begun or very early in a patient's treatment course. The specific elements of radiation oncology peer review are tailored to radiation treatment plans in particular, yet the process of completing a qualitative peer-reviewed evaluation of a patient's treatment plan has broad applicability to other fields of medicine. This analysis demonstrated that multiple stakeholders derive a wide range of benefits from the process and laid the groundwork for the next steps in optimizing the process to improve outcomes for both patients and provider stakeholders.

## Conclusion

This study demonstrated the majority of participants agreed peer review is an important process to improve radiation oncology QA despite a variation in the perceived benefits of peer review. The primary function of peer review was identified to align clinical practice between providers within institutional guidelines and nonphysician participant education. Future processes to assist in communication through institutional planning guidelines, prospective peer review to allow for plan modification, and feedback on plan modification to peers were identified as beneficial to peer review discussions and improving plan revisions.

## Disclosures

None.

## Appendix Supplementary materials

PRO Appendix A Image, application 1

New Supplemental Table 1 9_27_24 Image, application 2

## Footnotes

Supplementary material associated with this article can be found in the online version at doi:10.1016/j.adro.2024.101708.

## References

[1] Hong DS, Boike T, Dawes S (2021). Accreditation program for excellence (APEx): A catalyst for quality improvement. Pract Radiat Oncol.

[2] American College of Radiation Oncology. ACRO Accreditation Manual. Accessed January 1, 2024.https://cdn.ymaws.com/acro.org/resource/resmgr/files/accreditation/acro_accreditation_manual_se.pdf

[3] Katie Albus (2023). https://accreditationsupport.acr.org/support/solutions/articles/11000062794-site-surveys-radiation-oncology-revised-5-2-2023.

[4] Marks LB, Adams RD, Pawlicki T (2013). Enhancing the role of case-oriented peer review to improve quality and safety in radiation oncology: Executive summary. Pract Radiat Oncol.

[5] Caissie A, Rouette J, Jugpal P (2016). A pan-Canadian survey of peer review practices in radiation oncology. Pract Radiat Oncol.

[6] Ohri N, Shen X, Dicker AP, Doyle LA, Harrison AS, Showalter TN. (2013). Radiotherapy protocol deviations and clinical outcomes: a meta-analysis of cooperative group clinical trials. J Natl Cancer Inst.

[7] Fairchild A, Straube W, Laurie F, Followill D (2013). Does quality of radiation therapy predict outcomes of multicenter cooperative group trials? A literature review. Int J Radiat Oncol Biol Phys.

[8] Brunskill K, Nguyen TK, Boldt RG (2017). Does peer review of radiation plans affect clinical care? A systematic review of the literature. Int J Radiat Oncol Biol Phys.

[9] Albert AA, Duggar WN, Bhandari RP (2018). Analysis of a real time group consensus peer review process in radiation oncology: an evaluation of effectiveness and feasibility. Radiat Oncol.

[10] van der Veer SN, de Keizer NF, Ravelli AC, Tenkink S, Jager KJ. (2010). Improving quality of care. A systematic review on how medical registries provide information feedback to health care providers. Int J Med Inform.

[11] Eden AR, Hansen E, Hagen MD, Peterson LE. (2018). Physician perceptions of performance feedback in a quality improvement activity. Am J Med Qual.

[12] Ezzell G, Chera B, Dicker A (2018). Common error pathways seen in the RO-ILS data that demonstrate opportunities for improving treatment safety. Pract Radiat Oncol.

[13] Hennink MM, Kaiser BN, Marconi VC. (2017). Code saturation versus meaning saturation: How many interviews are enough?. Qual Health Res.

[14] ASTRO, Safety Is No Accident. Accessed January 1, 2024. https://www-astro-org.webpkgcache.com/doc/-/s/www.astro.org/astro/media/astro/patient%20care%20and%20research/pdfs/safety_is_no_accident.pdf.

[15] Surucu M, Bajaj A, Roeske JC (2019). The impact of transitioning to prospective contouring and planning rounds as peer review. Adv Radiat Oncol.

[16] Kut C, Chang L, Hales RK (2023). Improving quality metrics in radiation oncology: implementation of pretreatment peer review for stereotactic body radiation therapy in patients with thoracic cancer. Adv Radiat Oncol.

[17] Tchelebi LT, Kapur A, Chou H, Potters L. (2023). A decade of prospective peer review: Impact on safety culture and lessons learned in a multicenter radiation medicine department. Pract Radiat Oncol.

[18] Hesse J, Chen L, Yu Y (2022). Peer review of head and neck cancer planning target volumes in radiation oncology. Adv Radiat Oncol.

[19] Farris JC, Razavian NB, Farris MK (2023). Head and neck radiotherapy quality assurance conference for dedicated review of delineated targets and organs at risk: Results of a prospective study. J Radiother Pract.

[20] Hughes RT, Tye KE, Ververs JD (2022). Virtual radiation oncology peer review is associated with decreased engagement and limited case discussion: Analysis of a prospective database before and during the COVID-19 pandemic. Int J Radiat Oncol Biol Phys.

[21] McClelland S, Amy Achiko F, Bartlett GK (2021). Analysis of virtual versus in-person prospective peer review workflow in a multisite academic radiation oncology department. Adv Radiat Oncol.

[22] Ali N, Schreibmann E, Kayode O (2024). Implementation of a novel chart rounds application to facilitate peer review in a virtual academic environment. Adv Radiat Oncol.

[23] Li Q, Wright J, Hales R, Voong R, McNutt T. (2022). A digital physician peer to automatically detect erroneous prescriptions in radiotherapy. NPJ Digit Med.

[24] Lawrence YR, Whiton MA, Symon Z (2012). Quality assurance peer review chart rounds in 2011: a survey of academic institutions in the United States. Int J Radiat Oncol Biol Phys.

