# Supplementary material for: Provider Practices and Perceived Barriers and Facilitators in Improving Quality Practices in Radiation Oncology Peer Review
Source: Adv Radiat Oncol. 2025 Jan 8;10(3):101708. doi: 10.1016/j.adro.2024.101708 (PMC11836489; doi:10.1016/j.adro.2024.101708)
Supplement: PRO Appendix A [file mmc1.docx]

**A.1. Interview guide**

- Welcome
- Introduction of moderator and assistants
- Goal of the study
- The goal of this interview is to understand and identify ways that we can improve decision-making and communication between providers during the peer review process. Peer review or chart rounds is a conference that reviews pertinent patient history as well as re-evaluation of case-specific qualitative decisions including: planning methods, prescription dose, target volumes, dose-volume histogram, patient setup, and minor or major changes made during a patient’s treatment course by a multidisciplinary team
- The results of this interview will be used to create a tool that will help the radiation oncology team to better communicate and catch errors during the radiation treatment planning process.
- You have been asked to participate in this interview because you were identified as a health care provider who is involved in the peer review process
- Guidelines
- The researchers for this project have obtained institutional review board (IRB) approval for this study.
- Reminder about audio-recording (this is included in the waiver of documentation of consent document).
- Remind participants to not disclose identifying information (this is included in the waiver of documentation of consent document).
- Icebreaker activity
- Questions

1. What is your role in the peer review process?
2. What is your department’s process for radiation peer review, how often is it performed? When are patients reviewed during their radiation oncology treatment course? Who is present at your peer review?
3. Is your peer review site/technique specific or generalized for the whole department?
4. What is reviewed during peer review this can include (management plan, prescription, contours, treatment plan, clinical trial eligibility)
5. When does peer review occur during a patient’s plan (Prior to treatment start, early or within 25% of treatment period, anytime in the treatment course, or after treatment has been completed)
6. From a Likert scale ranging from (0-10, eg, ‘not important’, 0 and ‘extremely important’, 10). What would you rate the importance of peer review. What is the greatest value from peer review discussion from the Likert scale (Detection of medical error, Improvement of treatment planning processes, conformity of plans with existing policy, reduce variation in practice, reduce treatment incidents, education)
7. What do you think the rate of errors/variances detected during peer review? What is the rate of any type of error? What is the rate of clinically significant error?
8. How do you document errors and what is your system to reaudit a patient when change in management is discussed?
9. Do you think peer review is an important process of radiation oncology treatment quality
10. De-identified Case Studies & Questions
    - Case #1: Missing clip in APBI
    - Case #2: Thoracic Re-irradiation
    - Case #3: Wrong Iso?
    - FMEA Outcome severity scale
11. Low (minimal resident harm)
12. Moderate (short-term resident harm)
13. Severe (permanent or long-term harm)
14. Fatal (death)
    - FMEA Probability
15. Remote: <1 failure in 500,000
16. Low: 1 failure in 5000
17. Moderate 1 failure in 500
18. High: 1 failure in 50
19. Very high: 1 failure in 5
    - Please describe if recommendation will affect your practice
    1. Change in process/will be made for this plan
    2. Change not made now but will institute in future plans
    3. Not clinically relevant/stylistic change
    - If recommended change was not performed please select contributing factors to your decision or describe additional decisions:
    1. Patient Delay
    2. Increased Time/Resource burden
    3. Difficulty further optimizing plan
    4. Patient specific considerations (i.e performance status/social economic factors)
    5. Recommendation does not affect plan effectiveness/patient harm
    6. No data supporting recommendation

5. Summary and closing remarks
