# Supplementary material for: Provider Practices and Perceived Barriers and Facilitators in Improving Quality Practices in Radiation Oncology Peer Review
Source: Adv Radiat Oncol. 2025 Jan 8;10(3):101708. doi: 10.1016/j.adro.2024.101708 (PMC11836489; doi:10.1016/j.adro.2024.101708)
Supplement: New Supplemental Table 1 9_27_24 [file mmc2.docx]

Table E1: Qualitative results: Facilitators and Barriers for Peer Review Mediated Plan Changes

| **Theme** | Participant Profession | Descriptive and Illustrative Quotes |
| --- | --- | --- |
| **Facilitators:**  Improve Workflow and Planning Metrics | Attending Physician | “In our group this is a case that would ideally have been flagged for pre-treatment review […] and there would still be a little bit of time to make a change.” |
| **Facilitators:**  Technological and Personnel Support | Radiation Therapist | “There could be additional kind of workflows or safety check, like an actual form that's filled out rather than just believing it was done.” |
|  | Dosimetrist | “I'm going to go back to the team and ask them to verify that what I've done is correct. When I do that, I will copy the contour and put my initials after it because I want verification.” |
|  | Peer Review Software Developer | “[Peer review should] be able to give feedback individually, or in the most convenient time and place for the reviewer to ensure that we lower the barriers to peer review being done. There should be the ability to go through all the patients that should be reviewed without hiccups and technical problems, and having to switch between too many different software platforms” |
| **Barriers:** Lack of Communication | Radiation Therapist | “I don’t really feel like participation is really happening, […] you’re like sitting there waiting for someone to respond. And you don’t really know who is paying attention.” |
|  | Dosimetrist | “I know a lot of times people might not have all the clinical information. In that chart round, the individual that is responsible for that patient may not be there” |
| **Barriers:** Lack of Supporting Data | Attending Physician | “I was pushing out of the mandible, and the recommendation was to go soft on the mandible. Pull back on the coverage. That was the modification I made. I think it's stylistic, you know, almost there's much less of a right or wrong.” |
|  | Dosimetrist | “I think one of the things that needs to be asked is the plan clinically acceptable? […] If you’re talking about a 1, 2, 3% change, is it worth that time and affecting the people down the line?” |
| **Barriers:** Time Constraints | Dosimetrist | “You know how many meetings we've got nowadays. You can't take everybody out, and they have 2 to 3 dosimetrists in the meeting. There is not much that we can help other than present the plan” |
|  | Dosimetrist | “So now, where you had three days to plan something, you are now down to hours and that’s very stressful […] that can cause more errors instead of less.” |
|  | Attending Physician | “You know it's a real investment in time. All of these very busy and very highly trained minds all putting multiple hours a week into this process.” |
|  | Attending Physician | “But the reason to let this go through oftentimes is going to be time. Replanning is going to delay this patient. […] There’s not enough time to make those changes and that would be the reason not to do anything. And I think that happens more often than we’re willing to admit.” |
|  | Attending Physician | “This plan has already been generated. You’re resistant to any change at this point. You feel bad for your planner for having to redo all the work that they already did, because you made a silly mistake.” |
| **Barriers:** Documentation | Attending Physician | “Sometimes we focus a lot on administrative bookkeeping stuff. [...] Not to say that isn’t important, but then we miss the big picture” |
|  | Attending Physician | “I am bothered by so much documentation and meetings. [...] Because I'm the one reaching out for help [during peer review] I probably wouldn't end up document it, and I would just do what I need to take care of my patients.” |
